# COVID-19 outbreak in long-term care facilities from Spain. Many lessons to learn

**DOI:** 10.1371/journal.pone.0241030

**Published:** 2020-10-27

**Authors:** Marta Mas Romero, Almudena Avendaño Céspedes, María Teresa Tabernero Sahuquillo, Elisa Belén Cortés Zamora, Cristina Gómez Ballesteros, Victoria Sánchez-Flor Alfaro, Rita López Bru, Melisa López Utiel, Sara Celaya Cifuentes, Luz María Peña Longobardo, Antonio Murillo Romero, Laura Plaza Carmona, Borja Gil García, Ana Pérez Fernández-Rius, Rubén Alcantud Córcoles, Belén Roldán García, Luis Romero Rizos, Pedro Manuel Sánchez Jurado, Matilde León Ortiz, Pilar Atienzar Núñez, Alicia Noguerón García, María Fe Ruiz García, Rafael García Molina, Juan de Dios Estrella Cazalla, Juan Oliva Moreno, Pedro Abizanda

**Affiliations:** 1 Department of Geriatrics, Complejo Hospitalario Universitario of Albacete, Albacete, Spain; 2 CIBERFES, Ministerio de Economía y Competitividad, Madrid, Spain; 3 Department of Economic Analysis and Finance, Universidad de Castilla-La Mancha, Toledo, Spain; 4 Long-term Care Facilities Coordination, Complejo Hospitalario Universitario of Albacete, Albacete, Spain; 5 Vasco Núñez de Balboa Facility, Albacete, Spain; University of Technology Sydney, AUSTRALIA

## Abstract

**Background/Objectives:**

To analyze mortality, costs, residents and personnel characteristics, in six long-term care facilities (LTCF) during the outbreak of COVID-19 in Spain.

**Design:**

Epidemiological study.

**Setting:**

Six open LTCFs in Albacete (Spain).

**Participants:**

198 residents and 190 workers from LTCF A were included, between 2020 March 6 and April 5. Epidemiological data were also collected from six LTCFs of Albacete for the same period of time, including 1,084 residents.

**Measurements:**

Baseline demographic, clinical, functional, cognitive and nutritional variables were collected. 1-month and 3-month mortality was determined, excess mortality was calculated, and costs associated with the pandemics were analyzed.

**Results:**

The pooled mortality rate for the first month and first three months of the outbreak were 15.3% and 28.0%, and the pooled excess mortality for these periods were 564% and 315% respectively. In facility A, the percentage of probable COVID-19 infected residents were 33.6%. Probable infected patients were older, frail, and with a worse functional situation than those without COVID-19. The most common symptoms were fever, cough and dyspnea. 25 residents were transferred to the emergency department, 21 were hospitalized, and 54 were moved to the facility medical unit. Mortality was higher upon male older residents, with worse functionality, and higher comorbidity. During the first month of the outbreak, 65 (24.6%) workers leaved, mainly with COVID-19 symptoms, and 69 new workers were contracted. The mean number of days of leave was 19.2. Costs associated with the COVID-19 in facility A were estimated at € 276,281/month, mostly caused by resident hospitalizations, leaves of workers, staff replacement, and interventions of healthcare professionals.

**Conclusion:**

The COVID-19 pandemic posed residents at high mortality risk, mainly in those older, frail and with worse functional status. Personal and economic costs were high.

## Introduction

Long-term care facilities (LTCFs) are high-risk settings for respiratory disease outbreaks, including Covid-19. The first published COVID-19 outbreak in a LTCF included 130 residents and 170 staff. In the facility, after the first detected case, 77.7% of the residents and 29.4% of the healthcare personnel were confirmed for COVID-19. Hospitalization rates for facility residents and staff were 54.5% and 6.0%, respectively, and the case fatality rate for residents was 33.7% [1]. Thereafter, data from four UK nursing homes has been published including 394 residents and 74 staff, reporting an excess mortality of 203% in a two-month period compared with previous years [2].

Older adults are the population group accounting for the large majority of severe COVID-19 cases, hospitalizations and deaths [3]. The World Health Organization (WHO) has just issued guidance for LTCFs, stating that they must take special precautions to protect their residents, employees, and visitors [4, 5]. The document recognizes that infection prevention and control activities may affect the mental health and well-being of residents and staff, especially the use of personal protective equipments (PPE) and restriction of visitors and group activities, and describes guidance for prevention and response strategies, case reporting, and actions for minimizing the effect of prevention on mental health of residents, employees, and visitors [4]. The Centers for Disease Control and Prevention (CDC) have published the document “Preparedness checklist for Nursing Homes and other LTC-settings”, including recommendations for planning and decision making, and the elements that should be included in a written plan [5]. The Centers for Medicare & Medicaid Services (CMS), responsible for ensuring the health and safety of US nursing home residents by enforcing the standards required to attain their highest level of well-being, have also provided guidance to nursing homes to help control and prevent the spread of the virus [6]. Finally, the American Geriatrics Society recently published a policy brief statement regarding COVID-19 and Nursing Homes [7].

At the beginning of March 2020, Spain presented one of the worst COVID-19 outbreaks throughout the world, with 342,813 infected people (10% of Europe confirmed cases, the second country with more cases after Russia) and 28,617 official deaths until 2020 August [8]. The impact was uneven throughout the different regions of the country, and Castilla–La Mancha, the region where Albacete is located in the center of Spain, was among the worst affected areas. Many of the 5,417 Spanish LTCFs were dramatically affected, some of them in precarious conditions, and lack of PPE or staff training was reported by national and international media [9–12]. Three in every four LTCFs in Spain were privately run and many residents had some of their costs publicly funded. The fees received by the institutions had not changed for a long time, a result of years of austerity in Spain, and many private facilities had to make cuts to make a profit, and some lacked equipment even in normal times, while many operated with minimum staff. On March 24, the Spanish Minister of Health published the document “Prevention and control guidelines against COVID-19 in nursing homes and other residential social services centers” [13], that presented the organizational rules and policies for the LTCFs during the pandemics, including the ability of the local authorities to intervene the governance and health care of the institutions at risk.

**Fig 1:**
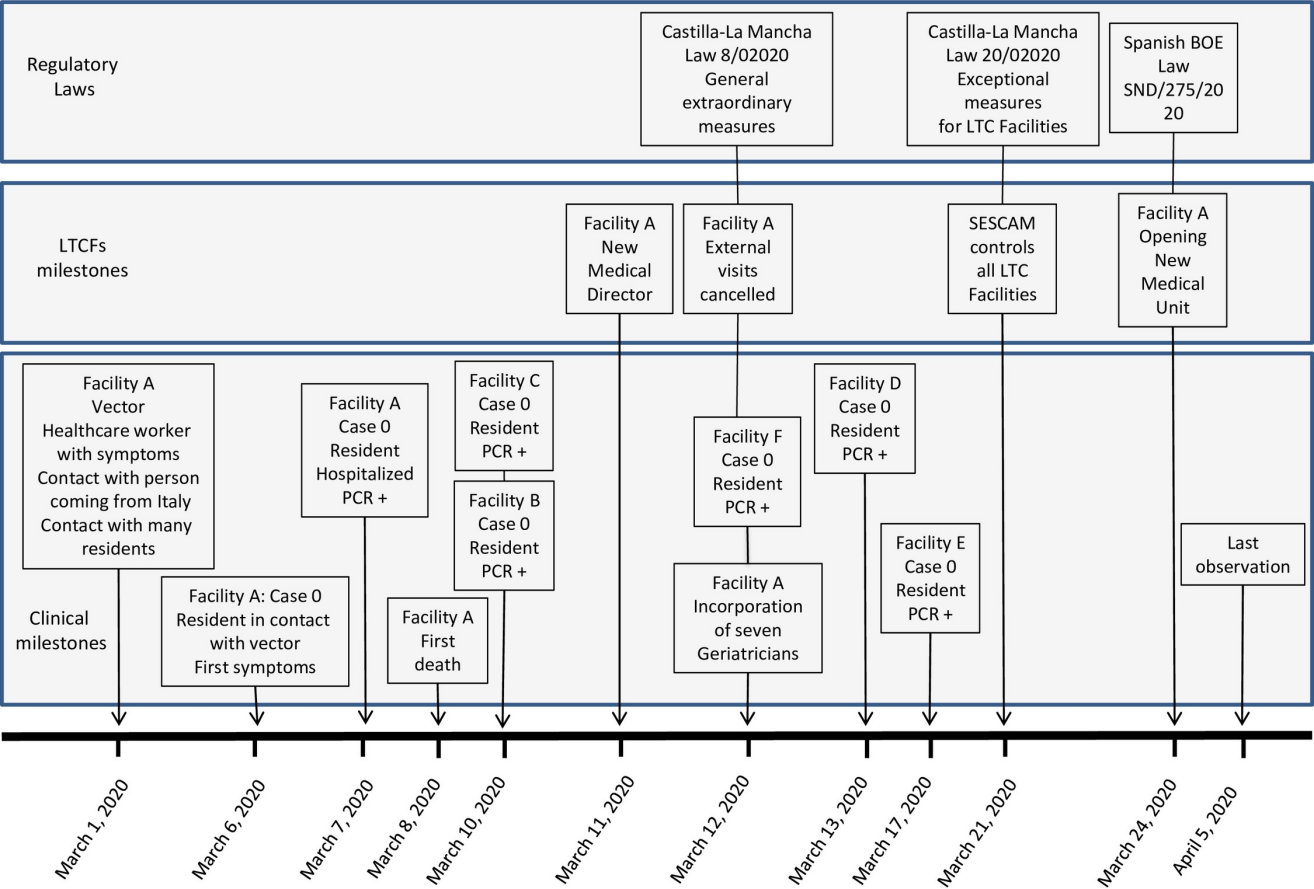
Timeline showing long-term care facilities outbreak in Albacete. PCR: Polymerase Chain Reaction. LTCF: Long-term care facilities. SESCAM: Healthcare Service of Castilla-La Mancha region. BOE: Boletín Oficial del Estado (Spanish Government bulletin for Laws).

On March 7, at the beginning of the Spanish COVID-19 outbreak, a first COVID-19 resident was detected in the LTCF A, Albacete, Spain. There were no protocols for epidemics, PPE were very scarce, and healthcare and non-healthcare staff were not trained. At that time, many of the workers were with symptoms at home. There was only a 9-bed medical unit with oxygen installation that was full from the first day, and most of the recommendations published by the CDC were not set up [4]. Health authorities were informed, the residence was closed to external visitors, and the geriatrics department from the reference hospital, took medical control of the facility. Fig 1 presents the timeline of the outbreak.

Case investigation, isolation of residents, and measures of infection prevention and control were immediately implemented. Contact tracing was difficult because of the high mobility of the residents previous to the outbreak, although families and caregivers were informed. Residents were classified for management depending on their basal characteristics. Residents were transferred to the hospital only if they had clear indication for intensive care unit (ICU) management, because of hospital collapse. Complete medical care, except invasive or non-invasive ventilation, was delivered in the 9-bed medical unit, in a new 12-bed medical unit installed in the assembly hall, or at the resident´s rooms of the facility. Hospitalization rates were very small, mainly in the first outbreak days, while the assessing of all the residents was completed. COVID-19 polymerase chain reaction (PCR) tests were only available at the beginning of the outbreak for patients needing hospitalization.

The publication of McMichael et al. describes perfectly the epidemiology of the outbreak in a facility [1]. However, the clinical characteristics of the residents, functional situation, frailty, mental status, nutritional situation, comorbidity, and treatments previous to the outbreak, outcomes and costs, were not analyzed, and could be relevant data in order to understand how COVID-19 affects institutions. Furthermore, there is a need for real epidemiology, and syndromic surveillance studies may help in identifying the overall burden and the attack rates in specific populations like those in institutions [14].

## Materials and methods

The main objective of the study was to analyze mortality, costs, residents and personnel characteristics, in six LTCFs during the outbreak of COVID-19 in Spain. The residents and staff of the facility A compose the main study population, with 198 residents and 190 workers (1 director, 2 physicians covering 64 hours/week, 12 nurses, 75 assistant nurses, 1 physiotherapist, 1 occupational therapist, 1 psychologist, 47 cleaning and catering services, 14 cooks, 36 other jobs), 106 with public contracts and 84 with private contracts. The facility has 3 residency floors with 125 bedrooms for both dependent and independent older adults, gym, therapy room, assembly hall, common rooms, day center, medical unit, pharmacy, dining room and adapted bathrooms. The medical unit is prepared for 9 residents with special needs.

We have also included epidemiological data of five more facilities in Albacete with a final number of 1,062 residents in order to external validate the outcome results, identified with the letters B, C, D, E, and F. In global, these six LTCFs account for more than 90% of all the residents of Albacete (175,000 inhabitants). Initially, data from March 6 until April 5 were collected from the six facilities and thereafter from March 6 until June 5, but only in facility A complete clinical data were obtained. Epidemiological data included total number of residents, date of the first case, number of confirmed cases (positive PCR), probable cases (typical symptoms and confirmed contact), mortality rate during the first and the first three months of pandemic, and mortality during the same periods in 2019.

Two trained geriatric specialist nurses, one nutritionist, one economist, and three residents in geriatric medicine collected the data through personal interviews with residents and staff, telephone interviews with staff at home, and medical records review of the residents. All 198 residents were identified and assessed, but we could only identify 147 workers for complete assessment. Demographics and functional status with the Barthel index [15], Lawton index [16], FRAIL instrument [17], Functional Ambulation Classification [18], and the New Functional Classification [19] were determined. Cognitive status and nutritional situation with the Mini Nutritional Assessment Short Form (MNA-SF) [20] were assessed, and comorbidity was determined with the Charlson index [21]. Main chronic diseases and chronic treatments were retrieved from the medical records. COVID-19 symptoms and treatments were assessed by personal interviews and medical records reviews. Transfers to the emergency department (ED), hospitalization, X-ray and lab test acquisition, and transfers to the medical unit were evaluated.

Public funder perspective was used to identify, measure and value costs related to intervention in the 30 days considered. We estimated the costs of healthcare professionals directly involved in the care, taking into account the time devoted to the intervention in the facility A and their gross wages. Healthcare transportation, ED use and diagnostic test (X-ray and blood analytic test) costs were assessed using the Order of 11/17/2014, of the Regional Ministry of Health and Social Affairs [22]. These costs are probably infra-estimated because more lab tests were realized, although they were not well retrieved. Inpatient costs were estimated using the average costs of Related Diagnosis Group published by the Ministry of Health, Consumption and Social Welfare [23]. In relation to the medication that patients received, they were valued at acquisition cost. Only the medication provided to treat COVID-19 (hydroxychloroquine, lopinavir/ritonavir, acetylcysteine, azithromycin, and methylprednisolone) was assessed. We also estimated the costs associated with the labour days lost due to the COVID-19 within the workers of the facility A. We considered that the workers temporarily leaving the job because of COVID-19 disease were replaced by another worker with the same characteristics (same sex and same professional category). Thus, so as to value the replacement costs, we used the gross wages provided by the Salary Structure Survey performed by the National Statistics Institute [24], differentiating by sex and professional category. All costs were updated to 2020 using the Consumer Price Index, subgroup of hospital services, provided by the National Institute of Statistics [25]. Some resources as oxygen therapy, as well as gloves or masks and other protective materials were not included, as it was not possible to count all of it. Likewise, only part of the sick leave of the staff of the residence could be counted and not the total.

Differences between groups were determined using chi square tests and t-tests. Excess mortality for the first outbreak month (March) and for the period March-May was calculated as: mortality during the outbreak periods—mortality in the same periods of 2019/ mortality in the same periods of 2019 *100. All analyses were done in IBM SPSS Statistics 22 (IBM Corp. Armonk, NY).

Our research was conducted in accordance with the Helsinki statement regarding human research. The study was approved by the Ethics Review Committee of Albacete (“Comité de Ética en Investigación con medicamentos de Albacete”), record 2020/04/039, who agreed on consent waiver, following the “International Ethical Guidelines for Health-related Research Involving Humans” [26]. However, residents and health professionals were orally informed of the study, and anonymization was immediate to clinical data acquisition. Health professionals were asked to allow researchers to have access to their labor data, extracting them under anonymization.

## Results and discussion

Table 1 presents case and mortality results for the six LTCFs included in the study. The pooled mortality rate for the first month and three first months of the outbreak were 15.3% and 28.0%, and the pooled excess mortality for these periods was 564% and 315% respectively. Mortality was almost 10-fold higher when compared with that of the previous year. The percentage of probable COVID-19 infected residents was 33.6%.

**Table 1:** Epidemiology of COVID-19 in six long-term care facilities in Albacete (Spain) between March 6 and April 5.

	Facility	Facility	Facility	Facility	Facility	Facility	Pooled
	A	B	C	D	E	F	data
Number of residents by 06/03/2020	198	242	134	183	207	120	1,084
Date first case	07/03/2020	10/03/2020	10/03/2020	13/03/2020	17/03/2020	12/03/2020	-
PCR positive (confirmed cases)/ PCR total realized (n)	21/25	0/0	21/48	52/70	28/28	12/12	134/183
Typical symptoms + contact (probable cases)	84	68	45	41	74	52	364 (33.6)
Mortality from 06/03/2020 until 05/04/2020. n (%)	32 (16.2)	25 (11.4)	14 (10.4)	35 (19.1)	33 (15.9)	27 (22.5)	166 (15.3)
Mortality from 06/03/2020 until 05/06/2020. n (%)	39 (19.7)	72 (29.8)	28 (20.9)	50 (27.3)	59 (28.5)	55 (45.8)	303 (28.0)
Number of residents during 2019	245	604	155	302	278	145	1,729
Mortality 2019 n (%)	31 (12.7)	90 (14.9)	23 (14.8)	60 (19.9)	71 (25.5)	25 (17.2)	300 (17.4)
Mortality March 2019. n (%)	3 (1.5)	5 (2.1)	1 (0.7)	5 (2.7)	9 (4.3)	2 (1.7)	25 (2.4)
Mortality March to May 2019. n (%)	12 (5.9)	18 (7.4)	3 (2.2)	18 (9.8)	19 (9.2)	3 (2.5)	73 (6.9)

Table 2 presents the clinical characteristics of the residents in facility A, comparing those with or without symptoms, and those who died or survived. Probable infected patients were older, more frail, and with a worse functional situation than those without COVID-19. 68% of the residents presented at least one COVID-19 symptom, being the most common fever, cough and dyspnea. The most important clinical findings were pulmonary crackles and respiratory insufficiency. During the 1-month follow-up, 32 residents died in facility A. Those who died were also older, more frequently male, with worse functionality, and higher comorbidity. The symptoms with higher association with mortality were fever, dyspnea and confusion. From the total of the residents, 54 were moved to the medical unit of the facility, 25 were moved to the ED and 21 were hospitalized. In all these residents, both COVID-19 symptoms and mortality rates were higher, showing a high severity of the disease.

**Table 2:** Clinical characteristics of residents in facility A.

	N	Total sample	COVID-19 Symptoms		Mortality COVID-19	
	valid		Yes (n = 134)	No (n = 62)	Yes (n = 32)	No (n = 166)
		(n = 198)				
Age	198	81.9 (10.6)	82.8 (10.2)	80.1 (11.2)	86.2 (7.4)†	81.1 (11.0)†
Female sex	198	114 (57.6)	76 (57.6)	36 (58.1)	11 (35.5)†	67 (63.8)†
FAC	196	3.2 (1.7)	3.0 (1.8)*	3.7 (1.5)*	2.9 (1.8)	3.3 (1.7)
Barthel index	188	63.7 (30.8)	59.7 (30.6)*	71.4 (30.3)*	54.8 (33.4)	65 (30.3)
FRAIL instrument	140	2.0 (1.4)	2.3 (1.3)†	1.5 (1.3)†	-	-
Lawton index	134	1.3 (1.5)	1.2 (1.6)	1.4 (1.4)	-	-
New Functional Classification	181					
• Indep BADL robust		22 (11.1)	9 (7.6)*	12 (19.4)*	0 (0.0)*	22 (13.5)*
• Indep BADL prefrail		20 (10.1)	9 (7.6)*	11 (17.7)*	0 (0.0)*	20 (12.3)*
• Frail		7 (3.5)	4 (3.4)*	3 (4.8)*	0 (0.0)*	7 (4.3)*
• Mild dep BADL		64 (32.3)	42 (35.6)*	22 (35.5)*	5 (27.8)*	59 (36.2)*
• Mod dep BADL		25 (12.6)	21 (17.8)*	4 (6.5)*	4 (22.2)*	21 (12.9)*
• Sev dep BADL		43 (21.7)	33 (28.0)*	10 (16.1)*	9 (50.0)*	34 (20.8)*
MNA-SF	127	11.0 (2.9)	10.7 (3.6)	11.5 (1.6)	-	-
EAT-10	116	0.8 (1.5)	1.0 (1.8)	0.6 (0.9)	-	-
Geriatric syndroms						
• Disability in BADL	189	132 (66.7)	96 (76.2)*	36 (58.1)*	18 (75.0)	114 (69.1)
• Frailty	140	56 (28.3)	42 (49.4)†	14 (25.9)†	-	-
• Cognitive impairment	176	57 (28.8)	36 (32.7)	17 (28.8)	-	-
• Falls last month	184	22 (11.1)	17 (13.9)	5 (8.2)	3 (13.0)	19 (11.8)
• Immobility	196	34 (17.2)	29 (21.8)*	5 (8.1)*	8 (26.7)	26 (15.7)
• Urinary incontinence	194	124 (62.6)	93 (69.9)*	30 (50.8)*	24 (77.4)	100 (61.3)
• Fecal incontinence	189	62 (31.3)	50 (39.1)*	12 (20.3)*	15 (53.6)*	47 (29.2)*
• Dysphagia	188	28 (14.1)	21 (16.9)	6 (9.7)	7 (26.9)	21 (13.0)
• Malnutrition risk	126	68 (34.3)	43 (57.3)	24 (48.0)	-	-
• Pressure ulcers	195	12 (6.1)	9 (6.8)	3 (4.8)	1 (3.4)	11 (6.6)
• Visual impairment	193	119 (60.1)	77 (58.8)	41 (68.3)	22 (71.0)	97 (59.9)
• Auditive impairment	191	68 (34.3)	46 (35.7)	21 (35.0)	16 (55.2)*	52 (32.1)*
Number chronic diseases	197	6.3 (4.0)	6.2 (4.3)	6.7 (3.2)	5.7 (5.1)	6.4 (3.7)
Charlson index	188	2.1 (1.6)	2.0 (1.6)	1.9 (1.6)	3.0 (1.9)†	1.9 (1.5)†
• Hypertension	198	113 (57.1)	75 (56.0)	38 (61.3)	18 (56.3)	95 (57.2)
• Diabetes	198	52 (26.3)	36 (26.9)	16 (25.8)	12 (37.5)	40 (24.1)
• Dementia	198	44 (22.2)	29 (21.6)	14 (22.6)	8 (25.0)	36 (21.7)
• Cardiovascular disease	198	136 (68.7)	88 (65.7)	48 (77.4)	20 (62.5)	116 (69.9)
• COPD/Asthma	198	14 (7.1)	10 (7.5)	4 (6.5)	2 (6.3)	12 (7.2)
Number of drugs	198	8.7 (5.6)	8.8 (5.9)	8.7 (4.7)	7.7 (6.6)	8.9 (5.3)
Polypharmacy (> 5 drugs)	198	148 (74.7)	99 (73.9)	48 (77.4)	20 (62.5)	128 (77.1)
Chronic consumed drugs	198					
• ACE inhibitors		25 (12.6)	17 (12.9)	8 (12.9)	3 (9.4)	22 (13.3)
• ARB		47 (23.7)	28 (21.2)	19 (30.6)	4 (12.5)	43 (25.9)
• Hypotensors		115 (58.1)	77 (58.3)	38 (61.3)	16 (50.0)	99 (59.6)
• Statins		35 (17.7)	22 (16.7)	13 (21.0)	1 (3.1)*	34 (20.5)*
• Oral anticoagulants		30 (15.2)	17 (12.9)	13 (21.0)	5 (15.6)	25 (15.1)
• Antiagregants		52 (26.3)	36 (27.3)	15 (24.2)	7 (21.9)	45 (27.1)
• NSAIDs		15 (7.6)	13 (9.8)	2 (3.2)	0 (0.0)	15 (9.0)
• Hypoglycemiants		43 (21.7)	31 (23.5)	12 (19.4)	9 (28.1)	34 (20.5)
• Neuroleptics		41 (20.7)	23 (17.4)	18 (29.0)	8 (32.0)	33 (19.9)
• Benzodiacepines		67 (33.8)	44 (33.3)	23 (37.1)	7 (21.9)	60 (36.1)
• AChEI/Memantine		10 (5.1)	3 (2.3)†	7 (11.3)†	0 (0.0)	10 (6.0)
• Inhalatory drugs		44 (22.2)	27 (20.5)	17 (27.4)	9 (28.1)	35 (21.1)
• Proton pump inhibitors		82 (41.4)	58 (43.9)	23 (37.1)	11 (34.4)	71 (42.8)
• Fever	194		74 (55.2)		23 (74.2)‡	51 (31.3%)‡
• Cough	196		89 (66.4)		18 (56.3)	71 (43.3)
• Dyspnea	196		76 (56.7)		23 (71.9)‡	53 (32.3)‡
• Myalgia	196		19 (14.2)		5 (15.6)	14 (8.5)
• Confussion	196		36 (26.9)		16 (50.0)‡	21 (12.8)‡
• Odynophagia	196		18 (13.4)		2 (6.3)	16 (9.8)
• Headache	196		24 (17.9)		3 (9.4)	21 (12.8)
• Rhynorrhea	196		19 (14.2)		1 (3.1)	18 (11.0)
• Chest pain	196		6 (4.5)		1 (3.1)	5 (3.0)
• Diarrhea	196		33 (24.6)		5 (15.6)	28 (17.1)
• Nausea and vomiting	196		35 (26.1)		9 (28.1)	26 (15.9)
• Respiratory crackles	145		81 (60.4)		23 (82.1)‡	58 (49.6)‡
• Respiratory insuficiency	196		78 (58.2)		26 (81.3)‡	52 (31.7)‡
Move to the Facility medical unit	198	54 (27.3)	53 (39.6)‡	81 (1.6)‡	19 (59.4)‡	13 (21.1)‡
Move to the Emergency Department	198	25 (16.4)	25 (18.9)*	0 (0.0)*	11 (37.9)†	14 (11.4)†
Hospitalization	198	21 (10.6)	21 (15.7)†	0 (0.0)†	11 (34.4)‡	10 (6.0)‡
Diagnostic tests						
• X-ray	198	17 (8.6)	17 (12.7)†	0 (0.0)†	9 (28.1)‡	8 (4.8)‡
• Lab analysis	198	25 (12.6)	25 (18.7)‡	0 (0.0)‡	11 (34.4) ‡	14 (8.4) ‡
• Oxygen therapy	196		86 (64.2)		27 (84.4)‡	59 (36.0)‡
• Paracetamol/Metamizol	196		93 (69.4)		28 (87.5)‡	65 (39.6)‡
• Azithromycin	196		100 (74.6)		28 (87.5)‡	72 (43.9)‡
• Methylprednisolone iv	196		33 (24.6)		15 (46.9)‡	18 (11.0)‡
• Lopinavir/Ritonavir	196		46 (34.3)		13 (40.6)*	33 (20.1)*
• Hydroxychloroquine	196		59 (44.0)		18 (56.3)‡	41 (25.0)‡
• N-Acetylcisteine	196		56 (41.8)		11 (34.4)	45 (27.4)

All data are means (SD) or number of participants (%). FAC: Functional Ambulation Classification. BADL: Basic activities of daily living. MNA-SF: Mini-Nutritional Assessment Short Form. EAT-10: Eating Assessment Tool-10. COPD: Chronic Obstructive Pulmonary Disease. ACE: Angiotensin converting enzyme. ARB: Angiotensin receptor blocker. NSAIDs: Non steroidal anti-inflammatory drugs. AChEI: Acetyl-cholinesterase inhibitors. iv: intravenous.

* *p*<0.05

† *p*<0.01.

During the 1-month period analyzed, 65 workers leaved their work for medical reasons, mainly COVID-19 symptoms. These included 1 physician, 7 nurses, 43 assistant nurses and 14 cleaning and catering workers. We could not find the number of other personnel sick leaved because they were private workers. Under these outbreak conditions, the facility contracted 12 new nurses, 36 assistant nurses, and 21 cleaning and catering workers. In addition, some workers had to increase their working time due to difficulties in finding new persons. It was impossible to exactly know what these figures were. Table 3 presents the occupational and clinical characteristics of the 147 workers contacted in facility A. Those who leaved were more frequently healthcare workers, did their job in fixed time (mainly morning and night), had more chronic diseases, consumed more drugs, and presented more symptoms. 62.6% of the workers presented COVID-19 symptoms, being the most frequent cough, headache, myalgias, diarrhea, ageusia and anosmia. Most of them were only treated with paracetamol or metamizol, and the mean number of days of leave was 19.2.

**Table 3:** Clinical characteristics of 147 workers contacted in facility A.

	Total sample (n = 147)	Leave	
		Yes (n = 33)	No (n = 114)
Age	45.2 (10.8)	45.7 (11.2)	45.1 (10.8)
Female sex	120 (81.6)	25 (75.8)	95 (83.3)
Health care worker	103 (70.1)	29 (28.2)*	4 (9.1)*
Profesional category			
• Physician	2 (1.4)	1 (0.3)	1 (0.9)
• Nurse	12 (8.2)	2 (6.1)	10 (8.8)
• Assistant nurse	84 (57.1)	23 (69.7)	61 (53.5)
• Other healthcare	5 (3.5)	2 (6.1)	3 (2.6)
• Catering and cleaning	20 (13.6)	0 (0.0)	20 (17.5)
• Other non-healthcare	24 (16.2)	5 (15.2)	19 (16.7)
Working time			
• Morning	45 (30.6)	18 (54.5)‡	27 (23.7)‡
• Afternoon	32 (21.8)	6 (18.2)‡	26 (22.8)‡
• Night	6 (4.1)	4 (12.1)‡	2 (1.8)‡
• Rotatory	64 (43.6)	5 (12.1)‡	59 (51.8)‡
Days of leave	-	19.2 (7.5)	-
Contact with Covid residents			
• Close with PPE	23 (15.6)	4 (12.1)	19 (16.7)
• Close without PPE	93 (63.3)	22 (66.7)	71 (62.3)
• Casual without PPE	31 (21.1)	7 (21.2)	24 (21.1)
Visit to ED or FP	42 (28.6)	26 (78.8)‡	16 (14.0)‡
Number chronic diseases	0.6 (1.0)	0.9 (1.2)*	0.5 (0.9)*
Number of chronic drugs	0.6 (1.2)	1.2 (1.7)*	0.5 (1.0)*
COVID-19 Symptoms	92 (62.6)	32 (97.0)‡	60 (52.6)‡
• Fever	36 (24.5)	19 (57.6)‡	17 (14.9)‡
• Cough	57 (38.8)	23 (69.7)‡	34 (29.8)‡
• Dyspnea	29 (19.7)	12 (36.4)†	17 (14.9)†
• Myalgia	47 (32.0)	24 (72.7)‡	23 (20.2)‡
• Confussion	3 (2.0)	2 (6.1)	1 (0.9)
• Odynophagia	31 (21.1)	10 (30.3)	21 (18.4)
• Headache	56 (38.1)	23 (69.7)‡	33 (28.9)‡
• Rhynorrhea	18 (12.2)	5 (15.2)	13 (11.4)
• Chest pain	10 (6.8)	5 (15.2)*	5 (4.4)*
• Diarrhea	31 (31.4)	12 (36.4)†	18 (15.8)†
• Nausea and vomiting	27 (18.4)	16 (48.5)‡	11 (9.6)‡
• Ageusia	43 (29.3)	17 (51.5)†	26 (23.2)†
• Anosmia	44 (29.9)	16 (48.5)†	28 (24.6)†
• Oxygen therapy	1 (0.7)	-	-
• Paracetamol/Metamizol	71 (48.3)	29 (87.9)‡	42 (36.8)‡
• Azithromycin	8 (5.4)	4 (12.1)	4 (3.5)
• Methylprednisolone iv	2 (1.4)	1 (3.0)	1 (0.9)
• Lopinavir/Ritonavir	2 (1.4)	2 (6.1)†	0 (0.0)†
• Hydroxychloroquine	2 (1.4)	2 (6.1)†	0 (0.0)†
• N-Acetylcisteine	8 (5.4)	4 (12.1)	4 (3.5)

All data are means (SD) or number of participants (%). PPE: Personal protective equipment. ED: Emergency department. FP: Family Physician. iv: Intravenous.

* *p*<0.05

† *p*<0.01

‡ *p*<0.001.

Table 4 summarizes the main items of total cost, as well as the number of residents who received the indicated resources. The estimated costs of the time of the healthcare professionals who implemented the intervention (three geriatricians and two family doctors), and considering a daily dedication of 9 hours for each professional during the 30 days of the intervention, amounts to a total of € 47,317 (17.1% of the total). 25 patients required urgent medical transportation and attention in the emergency department. The estimated costs were € 18,480 (6.7% of the total). Some type of diagnostic test was performed in 25 patients (25 lab tests and 17 X-ray), which represented a total of € 1,963 (0.7% of the total). 21 patients were hospitalized with an estimated cost of € 156,099 (56.5% of the total). The cost of medicines amounted to € 2,674 (1.0% of the total). Additionally, 33 workers of the centre had to leave temporarily their job due to COVID-19, leading to 633 working days lost. This caused a total cost of € 49,748 (representing 18.0% of the total cost). Broadly, the total costs associated with the COVID-19 in the facility were estimated at € 276,281during the period considered (from March 6^th^ to April 5th).

**Table 4:** Costs of the intervention from March, 6^th^ to April 5^th^ 2020 in facility A.

Concept	Residents that received the resource	Costs (€)	Cost Weight
Healthcare professionals (geriatricians and family doctors)	198	47,317	17.1%
Hospitalization	21	156,099	56.5%
Health care transportation and Emergency Department consultation	25	18,480	6.7%
Diagnostic test (25 lab analysis and 17 X-Rays)	25	1,963	0.7%
Drugs	102	2,674	1.0%
Replacement of the facility staff	198	49,748	18.0%
Total resources	198	276,281	100%

## Discussion

Older adults are the highest risk-population for adverse outcomes during the COVID-19 pandemic. The overall mortality rate of 2.3%, rises up to 8% in those aged 70 to 79, and nearly 15% in those aged 80 and older [27]. Nevertheless, older adults in LTCFs have the highest mortality among all of them, with case fatality rates for residents up to 33.7% [1]. In the United States of America, nationwide, cases have occurred at more than 4,000 facilities, and nearly 51,000 cases and over 10,000 deaths have been reported. In addition, LTCFs account for over half of deaths in six states, with figures between 8% and 60% in states reporting data [28]. Our study, including 1,062 residents, confirms these findings with a pooled mortality rate for the first month and three first months of the outbreak of 15.6% and 28.5%, and a pooled excess mortality for these periods of 564% and 315% respectively, figures close to those observed in the UK, with an excess mortality of 203% in the first two months in four Nursing Homes [2]. Moreover, the mortality that occurred in the six facilities from 2020 March 6 until April 5, almost equals the mortality of the entire year 2019, being 10-fold higher when compared with the estimated monthly mortality rate of 2019.

A key challenge of our results could be the lack of clarity over COVID-19/non- COVID-19 suspected deaths, given asymptomatic residents or false-negative tests. There are three approaches to quantify COVID-19 related mortality in LTCFs. Including only deaths of residents who test positive, including deaths of residents with disease suspect based on symptoms or epidemiological issues, and excess deaths by comparison with figures in previous years [29]. It seems that comparing the deaths during the pandemic to deaths that have happened in previous years, named excess mortality, is the best way to estimate real mortality impact of COVID-19. This methodology also includes deaths that are indirectly linked to COVID-19, giving a holistic approach to the problem [29]. In addition, our results are free from the bias that could be associated with incomplete or partial data retrieval from National statistical offices, commonly responsible of data collection and report. Our data have been collected on-site by the research team, making them absolutely reliable.

The results of facility A show that those at higher risk of disease presentation are the oldest ones and the ones that are frail, with ambulation problems, or with disability in BADL. It is well known that disability [30] and frailty [31] are conditions independently associated with mortality. Also, it is known that frailty, as a pre-disability state, is the best independent predictor of adverse events in older adults, more than multimorbidity or polypharmacy [32, 33]. The low functional reserve of organs and systems associated with a low-grade pro-inflammatory state of frail older adults may account for these findings. A recent study in 9,395 nursing homes from the U.S. showed that 2,949 (31.4%) had documented COVID-19 cases. Larger facility size, urban location, non-chain status, and state were associated to an increased probability of having cases, while five-star rating, prior infection violation, Medicaid dependency, and ownership were not significantly related [34].

We could not find special associations with sex, any specific disease, multimorbidity count or polypharmacy. Available data from Italy, China, and UK, show that the most common comorbidities observed in COVID-19 patients are hypertension (63.1–74.7%), cardiovascular disease (50.5%), dementia (56.6%), and diabetes (22.0–30.5%), but some of these figures could be in relationship with LTCFs characteristics [2, 35]. We describe similar figures in our study. There was a tendency that those residents with worse nutritional status presented more COVID-19 symptoms, although we could not find associations with geriatric syndromes other than those associated to disability, frailty or functional impairment. Regarding special drug categories, only antidementia drugs and neuroleptics were more present in those without symptoms. We can not assume that these drugs or the physiopathology of dementia protect against the virus, and plausible explanations may be that residents with dementia are pauci-symptomatic or report less frequently the symptoms of the disease. Approximately, two thirds of the residents and the workers reported symptoms, but with different rates of presentation. While cough, dyspnea and fever were the most common among residents, cough, headache, myalgias, diarrhea, ageusia and anosmia were the most frequent among healthcare workers. These differences could be explained by data acquisition dates between residents and staff, underreporting in some residents, or age and sex differences. Similar figures were found in previous reports [2, 36].

The small number of residents moved to the emergency department or hospitalized was due to the Geriatricians intervention, which tried to manage as much residents in the facility, including move to the medical unit. A recent experience in Singapore has demonstrated that active measures can be effective in the spread of Covid-19 in LTCFs. These measures include that all patients with fever and respiratory symptoms are referred to acute hospitals in order to rule out COVID-19, that all residents admitted with acute respiratory infections are isolated in negative pressure rooms and tested once for COVID-19 if the clinical suspicion is low, or twice prior to transfer to a general ward, and also include the cohorting of patients with respiratory infections when necessary [37]. Another experience in Toronto, Canada, presents a hospital-nursing home partnership that was characterized in several phases: 1) engagement, relationship and trust-building; 2) environmental scan, team-building and immediate response; 3) early phase response; and 4) stabilization and transition period [38]. Authors state that it is not too late for health systems to regroup and restructure to help homes survive the surge of COVID-19 outbreaks.

The question whether hospitalization is indicated for older residents with COVID-19 and functional impairment, cognitive decline or multimorbidity, needs to be very carefully considered, because this population would prefer to die, not in an emergency room, hospital isolated room or in an intensive-care unit, but in their familiar environment [39]. Although facilites should restrict visitation of all visitors and non-essential healthcare personnel, exceptions could be done under certain compassionate care situations, such as an end-of-life [6]. Advance care planning should be encouraged in these residents in order to minimize harms and adequate the care to personal wishes.

The estimated costs of the outbreak in facility A have been clearly conservatively estimated. First, because there is not a group related to the diagnosis clearly adjusted to COVID-19. The consumption of resources due to hospitalizations is possibly higher than that indicated in our results. Secondly, as we have mentioned, there are resources that we have not been able to assess specifically (for example, oxygen therapy and protective and hygiene material). Regarding the replacement of professionals, we have only been able to have part of the information. Therefore, given the information gaps and given the conservative valuation choices made by the researchers, the economic impact in terms of resources used should be interpreted as a minimum value, being the real economic impact greater than the estimate. This leads to an important reflection for decision-makers on the need to coordinate health services more intensively with long-term care provided in facilities.

Our study has some limitations. The most important one is that it was not possible to obtain PCR confirmation for all the residents at the beginning of the outbreak, because initially it was only realized to hospital patients, and later, Albacete suffered a shortage of reactive. However, from an epidemiological point of view, simple counts of the number of confirmed cases can be misleading indicators of the epidemic’s trajectory if these counts are limited by problems in access to care or bottlenecks in laboratory testing, or if only patients with severe cases are tested [14]. In addition, clinicians were very strict and only considered probable cases when symptoms were typical and there have been a recent clear contact. Another limitation could be the external validity of our data due to differences in LTCFs or Health Systems from other countries. However, the mortality figures of our Facilities are similar to those described in the paper by McMichael et al. [1], and the inclusion of almost of the residents of our city favors external validity of our findings.

The main challenges for COVID-19 care in older adults are rapid testing, integration between social and healthcare services that is usually frail and fragmented, evaluation of the personal and social consequences of the isolation process, and implementation of geriatric interventions in order to prevent or reverse frailty and functional decline [40]. LTCFs need immediate support from policy makers and clinicians, and this need for clinical support will not end when the actual pandemic resolves [41]. Our work emphasizes the urgent need for preparing LTCFs in the entire world for future outbreaks, because older adults in institutions are the high-risk population. Frail and disabled residents should be identified as the highest risk ones.

## Supporting information

S1 File Minimal data set for residents. (SAV)

S2 File Minimal data set for workers. (SAV)
